# Body temperature patterns as a predictor of hospital-acquired sepsis in afebrile adult intensive care unit patients: a case-control study

**DOI:** 10.1186/cc12894

**Published:** 2013-09-12

**Authors:** Anne M Drewry, Brian M Fuller, Thomas C Bailey, Richard S Hotchkiss

**Affiliations:** 1 Department of Anesthesiology, Washington University School of Medicine, 660 S Euclid Avenue, St Louis, MO 63110, USA; 2 Departments of Anesthesiology and Emergency Medicine, Washington University School of Medicine, 660 S Euclid Avenue, St Louis, MO 63110, USA; 3 Department of Internal Medicine, Washington University School of Medicine, 660 S Euclid Avenue, St Louis, MO 63110, USA; 4 Departments of Anesthesiology, Surgery and Internal Medicine, Washington University School of Medicine, 660 S Euclid Avenue, St Louis, MO 63110, USA

## Abstract

**Introduction:**

Early treatment of sepsis improves survival, but early diagnosis of hospital-acquired sepsis, especially in critically ill patients, is challenging. Evidence suggests that subtle changes in body temperature patterns may be an early indicator of sepsis, but data is limited. The aim of this study was to examine whether abnormal body temperature patterns, as identified by visual examination, could predict the subsequent diagnosis of sepsis in afebrile critically ill patients.

**Methods:**

Retrospective case-control study of 32 septic and 29 non-septic patients in an adult medical and surgical ICU. Temperature curves for the period starting 72 hours and ending 8 hours prior to the clinical suspicion of sepsis (for septic patients) and for the 72-hour period prior to discharge from the ICU (for non-septic patients) were rated as normal or abnormal by seven blinded physicians. Multivariable logistic regression was used to compare groups in regard to maximum temperature, minimum temperature, greatest change in temperature in any 24-hour period, and whether the majority of evaluators rated the curve to be abnormal.

**Results:**

Baseline characteristics of the groups were similar except the septic group had more trauma patients (31.3% vs. 6.9%, *p *= .02) and more patients requiring mechanical ventilation (75.0% vs. 41.4%, *p *= .008). Multivariable logistic regression to control for baseline differences demonstrated that septic patients had significantly larger temperature deviations in any 24-hour period compared to control patients (1.5°C vs. 1.1°C, *p *= .02). An abnormal temperature pattern was noted by a majority of the evaluators in 22 (68.8%) septic patients and 7 (24.1%) control patients (adjusted OR 4.43, *p *= .017). This resulted in a sensitivity of 0.69 (95% CI [confidence interval] 0.50, 0.83) and specificity of 0.76 (95% CI 0.56, 0.89) of abnormal temperature curves to predict sepsis. The median time from the temperature plot to the first culture was 9.40 hours (IQR [inter-quartile range] 8.00, 18.20) and to the first dose of antibiotics was 16.90 hours (IQR 8.35, 34.20).

**Conclusions:**

Abnormal body temperature curves were predictive of the diagnosis of sepsis in afebrile critically ill patients. Analysis of temperature patterns, rather than absolute values, may facilitate decreased time to antimicrobial therapy.

## Introduction

Sepsis is a common, devastating disease that is the leading cause of death in critically ill patients [1]. It is recognized as a time-sensitive emergency, as patients stand the best chance for survival when effective treatment is delivered as early as possible [2-6]. Unlike other time-sensitive emergencies, such as myocardial infarction or stroke, the ability to detect the exact onset of sepsis is limited because there is no standard diagnostic test. Failure to accurately diagnose sepsis early can lead to delays in treatment and an unacceptable increase in morbidity and mortality [7].

Although an accepted definition of *sepsis *exists [8], diagnosis remains challenging because physicians must rely on nonspecific physiological symptoms and abnormal laboratory values to identify potentially septic patients. Two of the most classic signs--fever and elevated white blood cell (WBC) count--have repeatedly been shown to have poor sensitivity and specificity for the diagnosis of sepsis [9-15]. In critically ill patients, the diagnosis of hospital-acquired sepsis is particularly difficult because many of the classic signs and symptoms of sepsis may be masked by or mistakenly attributed to patients' underlying illnesses. Furthermore, some critical illnesses induce an immunosuppressive state that may prevent patients from mounting robust physiological responses to new infections [16,17]. Therefore, despite significant advances in our understanding of the pathophysiology of sepsis [18], our current diagnostic approach remains largely unchanged and inadequate [8,19].

**Figure 1:**
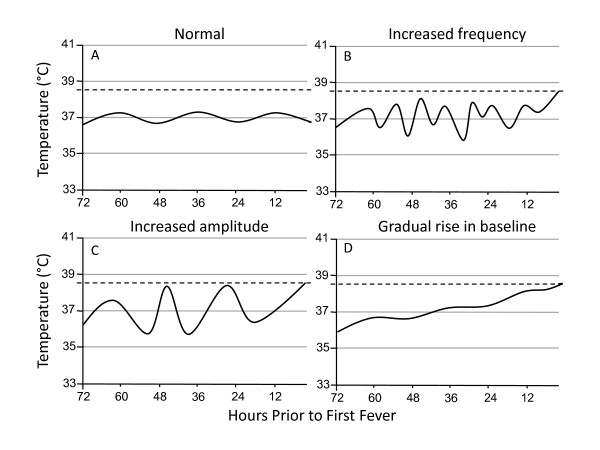
**Illustration of temperature pattern abnormalities observed prior to fever in septic patients**. The horizontal axes represent hours prior to the first fever in septic patients. The dotted lines denote a fever of 38.3°C. A normal body temperature pattern fluctuates diurnally by approximately 0.5°C around a mean of 37.0°C **(A)**. In septic patients, temperature patterns may exhibit increases in frequency **(B)**, increases in amplitude **(C) **or changes in baseline temperature **(D) **during the 72 hours prior to fever.

When evaluating patients for possible infection, physicians usually take into account the absolute values of certain vital signs to determine whether they meet a particular threshold indicative of infection. This is especially true with regard to body temperature; most physicians focus on the presence or absence of fever rather than following temperature trends. However, increasing evidence suggests that variability in the patterns of physiologic measures may be more specific for infection and may be an earlier indicator of sepsis than standard diagnostic criteria [20-23]. Baseline body temperature typically varies diurnally by approximately 0.5°C around a mean of 37.0°C in healthy individuals [24]. Members of our group have observed that fever in critically ill patients is often preceded by changes in this baseline body temperature pattern, and this led us to hypothesize that these abnormal temperature patterns may be an early indicator of sepsis. In a case series of 10 patients, we previously showed that critically ill patients with Gram-negative bacteremia developed subtle changes in their temperature patterns 24 to 72 hours prior to their first fever or the clinical diagnosis of sepsis [25]. Typical temperature pattern alterations included changes in amplitude, increases in frequency or increases or decreases in the baseline with loss of variability (see Figure 1).

The aim of this case-control study was to determine whether abnormal body temperature patterns, as typically measured in a clinical setting, can be used to distinguish between septic and nonseptic critically ill patients prior to the clinical diagnosis of sepsis. We hypothesized that the presence of temperature pattern abnormalities, as identified by visual examination of temperature curves prior to the onset of fever, would be significantly associated with the eventual diagnosis of sepsis. We also sought to identify characteristics of the temperature curves that could most definitively distinguish septic from nonseptic patients.

## Materials and methods

### Study design

We conducted a retrospective, observational case-control study and report its results in accordance with the Strengthening the Reporting of Observational Studies in Epidemiology (STROBE) guidelines [26]. Data collection and analysis were approved by the Human Research Protection Office at Washington University School of Medicine with waiver of informed consent.

### Study setting and population

This study was conducted in a 24-bed surgical ICU and a 19-bed medical ICU at a university-affiliated adult academic tertiary care hospital. Patients admitted to the ICU between 1 January 2010 and 31 December 2011 were evaluated for inclusion. Cases of sepsis were restricted to patients with proven infections, defined as a positive blood culture or positive bronchoalveolar (BAL) culture and the presence of at least two systemic inflammatory response syndrome (SIRS) criteria [19] within 24 hours of the time the culture was ordered. Cultures positive for common contaminants (for example, coagulase-negative *Staphylococcus *in one of two blood culture bottles, *Candida *in BAL cultures) were excluded.

To isolate ICU-acquired sepsis, only patients who developed infections more than 48 hours after admission to the ICU were included in the septic group. In accordance with guidelines promulgated by the Infectious Diseases Society of America, *fever *was defined as a temperature higher than 38.3°C [27]. Patients with cultures ordered or a fever higher than 38.3°C within 48 hours after admission to the ICU were excluded from the study because the goal was to use serial temperature measurements to diagnose sepsis prior to the clinical suspicion of infection.

The control group consisted of critically ill nonseptic patients admitted to the medical or surgical ICU who had an ICU length of stay more than two days but less than four days. Patients were excluded from the control group if they had any documented fever higher than 38.3°C in the 72-hour period prior to their discharge from the ICU or documented infections at any time during their ICU stay or within five days of discharge from the ICU.

Additional exclusion criteria for both groups included severe head trauma, requirement for continuous renal replacement therapy prior to the diagnosis of sepsis or the administration of scheduled nonsteroidal anti-inflammatory drugs (NSAIDs) and/or acetaminophen. These exclusion criteria were chosen to avoid including patients whose temperature patterns may have been altered due to external factors or neurologic injury.

### Study protocol

Septic cases were identified by querying the electronic medical records of patients admitted to the medical and surgical ICUs during the two-year study period for those who had positive blood or BAL cultures ordered more than 48 hours after admission to the ICU and who did not have any cultures ordered prior to this time. To identify control patients, the medical records were queried for patients who had ICU lengths of stay of two to four days and who did not have any positive cultures or treatment with antibiotics while in the ICU. Fifty patients were randomly selected from this cohort for potential inclusion in the control group. A detailed chart review of each of these patients was then performed by a study investigator, blinded to the temperature data, to identify those patients who met all inclusion and exclusion criteria.

### Data collection

#### Demographic, laboratory and outcome data

For all patients, baseline characteristics included age, sex, medical or surgical ICU admission, admission diagnosis, site of positive culture and organism and modified Acute Physiology and Chronic Health Evaluation II (APACHE II) score (excluding the Glasgow Coma Scale score). Admission diagnoses were categorized as cardiovascular disease, respiratory disease, gastrointestinal or renal disease, trauma or routine postoperative admission. The highest and lowest WBC counts were recorded during the period 72 hours prior to the diagnosis of sepsis (in septic patients) or discharge from the ICU (in control patients). A WBC count less than 4,000/μl or more than 12,000/μl was considered to be abnormal [19]. Process of care variables included mechanical ventilation requirement, vasopressor use and time from the end of the temperature curve to culture and initiation of antibiotics. Outcome measures included development of severe sepsis or septic shock, ICU length of stay and in-hospital mortality. *Severe sepsis *and *septic shock *were defined as previously described [19].

#### Generation of temperature plots

For each septic patient, the timestamp for the first clinical suspicion of sepsis was defined as the time of the first documented fever higher than 38.3°C, the time of the first culture from any site or the first dose of antibiotics, whichever came first. All documented temperature measurements were plotted for the period starting 72 hours and ending 8 hours prior to this timestamp. For each control patient, all documented temperature measurements were plotted for the period starting 72 hours and ending 8 hours prior to discharge from the ICU. A 72-hour time period was chosen to allow for collection of enough data for temperature pattern analysis [25].

#### Visual analysis of the temperature curves

The temperature curves from the septic and control patients were numbered and ordered by a random number generator. Seven surgical ICU intensivists (four anesthesiologists and three emergency medicine physicians), blinded to group assignment, independently and separately inspected the temperature curves and documented whether abnormal temperature patterns were present. Figure 1 shows samples of actual curves (size reduced for space) given to the evaluators for analysis. In instructions to the physician evaluators, abnormal temperature patterns were described as "increases in amplitude, changes in frequency, or loss of variability," but no specific numeric criteria were provided. No other clinical information about the patients was given to the evaluators. To measure intraevaluator reliability, the temperature curves were renumbered and reinspected by the same physicians approximately 16 weeks after the first review.

#### Temperature curve characteristics

For each temperature plot, the maximum and minimum temperatures over the 72-hour study period and the greatest change in temperature (maximum minus minimum temperature) over any 24-hour period was recorded. We also noted whether a majority (at least four of seven) of the reviewers rated the temperature curve as abnormal.

### Statistical analysis

Descriptive statistics, including mean (standard deviation), median (interquartile range) and frequency distributions were used to assess the characteristics of the patient cohort. Normality was assessed using the Kolmogorov-Smirnov test. To assess differences between groups, continuous and categorical variables were compared using an unpaired *t*-test, Wilcoxon rank-sum test, χ^2 ^test or Fisher's exact test, as appropriate.

Univariate logistic regression was used to model the odds of developing sepsis using each temperature curve characteristic and abnormal WBC count as independent variables. For categorical variables, the reference category for the odds ratio (OR) was absence of the condition. For continuous variables, the ORs reflect the increased odds of developing sepsis for a one-unit increase in the baseline variable. Additionally, we performed multivariate logistic regression analyses to model the odds of developing sepsis using trauma admission, requirement for mechanical ventilation and each temperature curve characteristic as independent variables. A multivariate model using abnormal WBC count, trauma admission and requirement for mechanical ventilation was also used for analysis. Collinearity diagnostics were evaluated to ensure independence of the independent variables. Multivariate models report ORs adjusted for all variables in the model.

Temperature curve evaluation was also assessed as a diagnostic test for sepsis. We calculated the sensitivity and specificity with 95% confidence intervals (CIs) of abnormal temperature curves (as determined by majority consensus) to diagnose early sepsis. Interobserver and intraobserver reliability were calculated using κ values for multiple raters. All statistical tests were carried out using commercially available software (SPSS version 20.0; SPSS, Inc, Chicago, IL, USA). All tests were two-tailed, and *P *values less than 0.05 were considered statistically significant.

## Results

**Figure 2:**
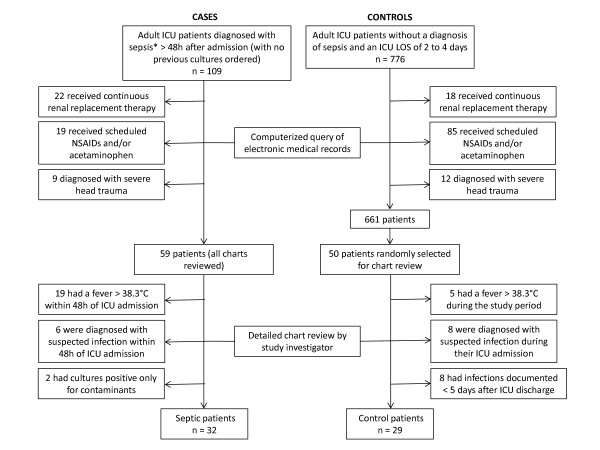
**Identification of septic and control patients**. *Sepsis was defined as the presence of a positive blood or bronchoalveolar lavage culture and at least two systemic inflammatory response syndrome criteria within 24 hours from the time the culture was ordered. LOS, length of stay; NSAID, nonsteroidal anti-inflammatory drug.

The initial computer query identified 59 potentially eligible surgical and medical ICU patients who had positive blood or BAL cultures ordered more than 48 hours after admission to the ICU. It also yielded 661 potentially eligible control patients from among whom 50 patients were randomly selected. Detailed chart reviews led to the exclusion of an additional 27 patients from the septic group and 21 patients from the control group. Of the patients excluded from the septic group, 19 had fevers higher than 38.3°C within 48 hours of admission to the ICU, 6 were being treated with antibiotics for suspected infection and 2 had cultures that were positive only for common contaminants (yeast in BAL cultures). Of the patients excluded from the control group, eight were being treated with antibiotics for a suspected infection while in the ICU, eight had positive cultures within five days after their ICU discharge and five had a fever higher than 38.3°C during the study period (Figure 2).

Table 1 gives the baseline characteristics and outcomes of the entire patient cohort. The groups were well-matched, except for more trauma patients (31.3% vs. 6.9%; *P *= 0.02) and more mechanically ventilated patients (75.0% vs. 41.4%; *P *= 0.008) in the septic group versus the control group. Septic patients had higher in-hospital mortality (28.1% vs. 6.9%; *P *= 0.03) and longer ICU lengths of stay (15.0 days vs. 4.0 days; *P *< 0.001) compared to the controls. Table 2 gives the characteristics of the septic group with respect to culture data and sepsis classification.

**Table 1:** Baseline characteristics and outcomes of the patients^a^

Characteristics and outcomes	Septic patients (*N *= 32)	Control patients (*N *= 29)	*P*
Mean age, years (± SD)	58.0 (19.5)	58.5 (13.6)	0.91
Males, *n *(%)	19 (59.4)	15 (51.7)	0.55
ICU type			
SICU, *n *(%)	19 (59.4)	12 (41.4)	0.16
MICU, *n *(%)	13 (40.6)	17 (58.6)	
Reason for ICU admission, *n *(%)			0.14
Cardiovascular disease	4 (12.5)	5 (17.2)	
Respiratory disease	6 (18.8)	9 (31.0)	
Gastrointestinal or renal disease	8 (25.0)	6 (20.7)	
Postoperative	4 (12.5)	7 (24.1)	
Trauma	10 (31.3)	2 (6.9)	
Mean APACHE II score (± SD)	15.7 (5.5)	15.0 (5.8)	0.64
Mechanical ventilation, *n *(%)	24 (75.0)	12 (41.4)	0.008
Vasopressors, *n *(%)	8 (25.0)	5 (17.2)	0.46
In-hospital mortality, *n *(%)	9 (28.1)	2 (6.9)	0.03
ICU LOS (days), median (IQR)	15.0 (9.5 to 22.0)	4.0 (3.0 to 4.0)	<0.001

^a^APACHE II, Acute Physiology and Chronic Health Evaluation II; IQR, 25% to 75% interquartile range; LOS, length of stay; MICU, medical intensive care unit; SD, standard deviation; SICU, surgical intensive care unit; WBC, white blood cell.

**Table 2:** Characteristics of septic patients (*N *= 32)

Characteristics	Data
Severe sepsis, *n *(%)	27 (84.4)
Septic shock, *n *(%)	21 (65.6)
Culture site, *n *(%)	
Blood	11 (34.4)
Respiratory	21 (65.6)
Type of organism, *n *(%)	
Gram-negative	15 (46.9)
Gram-positive	11 (34.4)
Mixed Gram-negative and Gram-positive	5 (15.6)
Fungal	1 (3.1)

**Figure 3:**
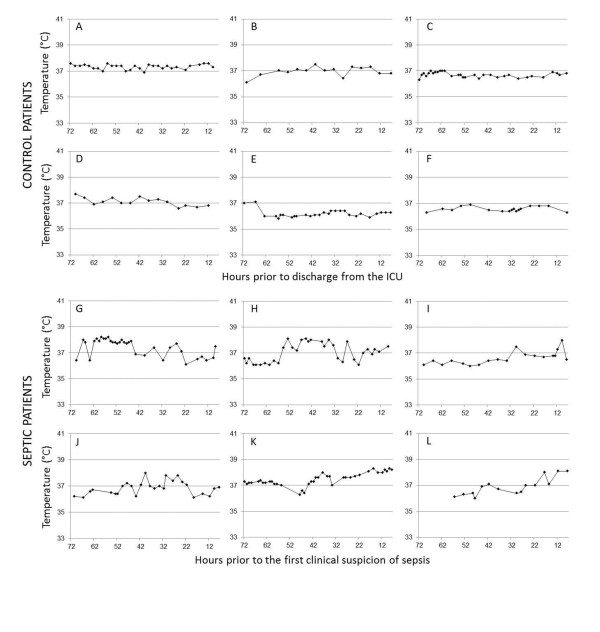
**Example temperature curves from afebrile septic and control patients**. The horizontal axes represent hours prior to the clinical suspicion of sepsis (in septic patients) or hours prior to discharge from the ICU (in control patients). The timestamp for the clinical suspicion of sepsis was defined as the time of the first fever, the time of the first culture (from any site) or the time the first antibiotic was ordered by the ICU medical staff, whichever came first. Note that the temperature plots end eight hours prior to the first clinical suspicion of sepsis.

Figure 3 shows sample temperature curves from the subset of septic and control patients who were correctly classified by the majority of the evaluators. Most (86.9%) of the temperature measurements in both groups were taken orally; 4.9% were axillary and 8.2% were bladder or esophageal. Because the inclusion criteria required a minimum ICU length of stay of only 48 hours, not every temperature curve had data points that spanned the entire 72-hour period. The median length of the plotted temperatures was not significantly different between the septic and control groups (70.0 hours (IQR = 66.5 to 72.0) vs. 70.0 hours (IQR = 59.5 to 71.0); *P *= 0.47). The frequency of temperature measurements in the control group was slightly lower than in the septic group (3.05 ± 0.86 hours vs. 3.58 ± 0.87 hours; *P *= 0.02).

The results of the univariate analysis of temperature curve characteristics are presented in Table 3. A majority of observers (at least four of seven) noted the presence of an abnormal temperature pattern in 22 (68.8%) of the septic patients and 7 (24.1%) of the control patients. Multivariate logistic analysis, used to adjust for differences in the percentages of trauma and mechanically ventilated patients in the two groups, demonstrated that an abnormal temperature curve was significantly associated with the subsequent diagnosis of sepsis (*P *= 0.017) with an adjusted OR of 4.43 (95% CI = 1.31 to 15.00). The greatest change in temperature during any 24-hour period was also significantly associated with sepsis (adjusted OR = 6.81 (95% CI = 1.35 to 34.25); *P *= 0.02), but maximum and minimum temperatures were not. The percentage of patients with WBC counts less than 4,000/μl or more than 12,000/μl was not statistically different between the two groups in either the univariate model (53.1% vs. 51.7%; *P *= 0.91) or the multivariate model when adjusted for trauma and mechanical ventilation (*P *= 0.43).

**Table 3:** Comparison of temperature curve characteristics and abnormal white blood cell count^a^

Characteristics	Septic patients (*n *= 32)	Control patients (*n *= 29)	Odds ratio^b^ (95% CI)	*P*	Adjusted odds ratio^c^ (95% CI)	*P*
Abnormal temperature pattern noted by majority of observers, *n *(%)	22 (68.8)	7 (24.1)	6.91 (2.23, 21.45)	.001	4.43 (1.31, 15.00)	.017
Maximum temperature (°C), mean (± SD)	37.8 (0.5)	37.4 (0.3)	5.85 (1.65, 20.8)	.006	3.43 (0.84, 14.03)	.087
Minimum temperature (°C), mean (± SD)	36.2 (0.4)	36.2 (0.2)	1.00 (0.22, 4.56)	.99	0.61 (0.10, 3.59)	.58
Greatest change in temperature within any 24-hour period (°C), mean (± SD)	1.5 (0.5)	1.1 (0.3)	10.95 (2.25, 53.35)	.003	6.81 (1.35, 34.25)	.02
Abnormal WBC count (<4,000/μl or >12,000/μl), *n *(%)	17 (53.1%)	15 (51.7%)	1.05 (0.39, 2.89)	.91	1.58 (0.51, 4.96)	.43

^a^CI, confidence interval; IQR, 25% to 75% interquartile range; SD, standard deviation. ^b^Univariate analysis using each temperature characteristic as the independent variable. ^c^Multivariate analysis using trauma, requirement for ventilation and temperature characteristic as independent variables. Reported odds ratios are adjusted for all variables in the model.

The diagnostic accuracy and reproducibility of temperature curve analysis to diagnose early sepsis is described in Table 4. The sensitivity was 0.69 (95% CI = 0.50 to 0.83) and the specificity was 0.76 (95% CI = 0.56 = 0.89). The multiple κ values for interobserver reliability was 0.50 (95% CI = 0.45 to 0.54), and the multiple κ value for intraobserver reliability was 0.60 (95% CI = 0.52 to 0.67).

**Table 4:** Temperature curve analysis as a diagnostic test for early sepsis^a^

Abnormal temperature pattern^b^	Septic (*n*)	Nonseptic (*n*)	Sensitivity (95% CI)	Specificity (95% CI)	Interrater κ^c^ (95% CI)	Intrarater κ^c^ (95% CI)
Present	22	7	0.69	0.76	0.50	0.60
Absent	10	22	(0.50 to 0.83)	(0.56 to 0.89)	(0.45 to 0.54)	(0.52 to 0.67)

^a^CI, confidence interval. ^b^Determined by the majority (at least four of seven) of the raters. ^c^κ statistic for multiple raters.

Some of patients had temperature curves that seemed to be more clearly identifiable by the evaluators as normal or abnormal. At least six of the seven observers agreed on thirty-eight (62.3%) of the sixty-one temperature curves. For this subset of patients, the sensitivity of an abnormal temperature curve to predict the clinical diagnosis of sepsis was 0.73 (95% CI = 0.50 to 0.88) and the specificity was 0.81 (95% CI = 0.54 to 0.95). Furthermore, in the subset of patients in whom all seven observers agreed (*n *= 25, 41.0%), the sensitivity and specificity were 0.72 (95% CI = 0.46 to 0.89) and 1.0 (95% CI = 0.56 to 1.0), respectively. For patients in the septic group, the median time from the end of the temperature plot to the first culture was 9.4 hours (IQR 8.0, 18.2) and median time to the first dose of antibiotics was 16.9 hours (IQR 8.4, 34.2).

## Discussion

ICU-acquired sepsis is an important cause of morbidity and mortality in critically ill patients [28-30]. Early aggressive treatment dramatically improves survival [2-7]. Thus, methods developed to diagnose sepsis earlier could have a significant impact in improving outcomes. The findings from this study provide further evidence that the examination of temperature variability could lead to an earlier diagnosis of sepsis in ICU patients.

Traditionally, temperature has been regarded as a dichotomous variable, with patients being categorized as febrile or not on the basis of absolute values. Evidence suggests, though, that body temperature pattern analysis may convey meaningful clinical information, regardless of whether patients meet the criteria for fever [25,31-33]. Varela *et al*. examined the role of temperature variability analysis in the prediction of survival in critically ill patients and found that the ability of temperature analysis to predict mortality was similar to that of Sequential Organ Failure Assessment scoring [31]. More recently, Papaioannou *et al*. used temperature variability analysis to distinguish septic shock from noninfectious SIRS in 22 critically ill patients with suspected ICU-acquired infections [33]. These previous investigations did not address the use of body temperature variability to predict the clinical onset of infection, however.

In our current study, we sought to determine whether body temperature patterns, as typically measured and recorded in an ICU setting, could aid in the diagnosis of sepsis before the appearance of other overt signs and symptoms of infection. We found that abnormal temperature patterns, as assessed by visual examination of readily available data, were identified more frequently in septic patients (prior to the diagnosis of sepsis) compared to controls. Compared to WBC count, temperature pattern assessment more accurately predicted impending sepsis. Likewise, because none of the patients in our study had temperatures higher than 38.3°C during the evaluated period, fever (as defined by this absolute value) would have been equally unhelpful in differentiating septic from nonseptic patients.

Additionally, we sought to identify specific characteristics of body temperature curves that could be used to distinguish afebrile septic patients from nonseptic patients. Although small (with a mean difference of only 0.4°C), the septic patients had statistically greater fluctuations in temperature over a 24-hour period compared to the noninfected patients. The septic patients also trended toward having higher maximum temperatures during the 72-hour period prior to diagnosis than the noninfected critically ill patients. In fact, fewer than 7% of control patients reached a temperature of 38.0°C, which was the median maximum temperature of patients in the septic group. Aside from these easily measurable changes, the temperature patterns of the septic patients exhibited other abnormalities that were more difficult to quantify. Frequently, they displayed a transition point at which a steadily oscillating baseline converted to a more erratic pattern (see Figure 3, patients H and I) or began to trend upward (patients K and L). In addition to having fluctuations of greater magnitude, the septic patients also exhibited more variable fluctuations, with small and large amplitudes interspersed within the same individual (see patients G, H and J).

Previous investigations of body temperature variability in septic patients found decreased variability to be associated with more severe disease [31-33]. In contrast, our study demonstrated that septic patients had greater and more irregular fluctuations in body temperature than nonseptic controls. These contradictory results may be partly due to differences in analytical techniques. Previous studies used statistical, frequency and geometric analyses to measure variability in very frequent, almost continuous temperature measurements [20,31-33]. However, our study consisted of visual assessment of relatively infrequent temperature measurements. Furthermore, previous investigations of body temperature variability focused on predicting disease severity in patients with existing multiple organ failure [31,32] or in patients who were already suspected of being infected [33]. None of the patients in our study, however, were clinically suspected of being septic during the time period of the temperature measurements. Therefore, we believe that timing is a critical element with respect to physiologic variability and hypothesize that body temperature variability is maintained prior to the clinical onset of sepsis, with loss of variability occurring as the disease progresses.

Thermoregulation is a complex process involving peripheral and central nervous system thermosensors, the spinal cord, the circulatory system and other autonomic effector sites [24]. Normally, this leads to body temperature fluctuations of approximately 0.5°C (with a range of 0.1°C to 1.2°C) around a mean of 37.0°C [24,34]. In critically ill patients, thermoregulatory processes are affected by multiple external and physiological variables, including vasoactive medications, sedation, mechanical ventilation and underlying disease processes [27]. Therefore, it is not surprising that, in our sample of patients, the mean maximum body temperature deviation over any 24-hour period for all patients approached the high end of the normal spectrum. Notably, though, patients with impending sepsis exhibited the highest fluctuations in temperature. We theorize that patients with early sepsis have low levels of circulating pathogenic mediators (for example, endotoxins) causing slight disruptions in normal homeostatic mechanisms prior to causing obvious clinical signs of infection. This hypothesis is supported by evidence showing increased levels of proinflammatory cytokines in septic, critically ill patients up to four days prior to the appearance of clinical symptoms [35].

The thermoregulatory response to sepsis varies among patients, and previous studies have suggested that the incidence of fever and/or hypothermia in infected patients may be influenced by age [12,36], comorbidities [14,37], source of infection [14] or type of organism [13]. In our study, we did not find any statistical differences in the temperature characteristics or patterns between patients with Gram-negative infections versus Gram-positive infections, patients with septic shock versus patients without shock or survivors versus nonsurvivors. Also, age did not act as a confounder when included in the multivariate analyses of temperature characteristics. However, future studies with larger numbers of patients may elucidate whether these factors affect temperature pattern features. In some patients, such as the elderly, who are less likely to develop a hyperthermic response to infection, subtle changes in body temperature pattern may in fact be more useful than fever as an indicator of sepsis.

This study has several limitations. Because of our narrow definition of sepsis, which included only patients with positive blood or BAL cultures, the total number of patients in the study was limited. We chose these strict criteria to avoid including patients erroneously diagnosed with sepsis based on clinical symptoms or growth of colonizing bacteria in sputum or urine cultures. Likewise, we excluded patients with ICU lengths of stay more than four days from the control group to prevent the inclusion of patients with undiagnosed subclinical infections, which were thought to be more likely in patients with prolonged ICU courses. Because the visual examination of temperature patterns to predict sepsis is a new concept, we aimed to make our septic and control groups as homogeneous as possible to best identify potential differences. This study also did not include the large group of patients who entered the ICU with a primary diagnosis of sepsis and recovered from the initial infection, only to develop secondary nosocomial infections later. This is a large population of ICU patients in whom examination of temperature variability may also be useful in the early detection of sepsis.

Also, as a retrospective study, the temperature measurement technique could not be standardized and the thermometers could not be calibrated. We found a statistically significant difference in mean maximum temperature fluctuation of only 0.4°C between the two groups in our study, which highlights the importance of accurate and precise temperature measurement. Core temperatures, preferred in treating critically ill patients, were recorded in only a small minority of the patients. Core temperatures tend to be slightly higher than peripheral temperatures, and the core-to-peripheral temperature gradient can fluctuate, depending on numerous external factors, such as ambient temperature, vasoactive medications and depth of sedation. Therefore, temperature curves consisting solely of core temperature measurements would likely provide a more accurate depiction of patients' thermoregulatory response to pathogens and could potentially improve the sensitivity and specificity of this technique. Additionally, the frequency of temperature measurements in our study varied significantly, which made it difficult to ensure that peak and trough temperatures and acute changes in pattern were fully appreciated in every patient. Nevertheless, the lack of standardization in our study allows the results to be more generalizable to typical ICU environments and to the data available to most physicians at the bedside.

Many critically ill patients develop noninfectious fevers. Studies suggest that less than half of fevers in the ICU are due to infections [12-15]. Our current study does not address the problem of distinguishing between infectious and noninfectious causes of fever. Our goal was to identify septic patients prior to the onset of clinical symptoms to facilitate earlier diagnosis and treatment. According to the American College of Critical Care Medicine and the Infectious Diseases Society of America, a temperature higher than or equal to 38.3°C in critically ill patients should reasonably prompt an evaluation for infection [27]. Our results suggest that a lower threshold for fever may facilitate earlier diagnosis of septic patients and that the magnitude of temperature fluctuations within a 24-hour period may be more meaningful than absolute temperature values. We are not suggesting that every critically ill patient with a temperature higher than 38.0°C or a temperature oscillation of more than 1.5°C receive invasive or expensive testing. These patients may, however, warrant more careful clinical evaluation because the morbidity and mortality of undiagnosed sepsis is great. Furthermore, assessment of the temperature pattern (including the frequency and amplitude of the oscillations and upward and downward trends) may also yield clinically useful information.

In this study, each of the temperature curves shown to the observers ended at least eight hours prior to the clinical diagnosis of sepsis. The median time from the end of the temperature plot to the first culture ordered by the ICU team was longer nine hours, and the median time to the first dose of antibiotics was close to seventeen hours. Moreover, many of the abnormalities identified on the temperature curves of the septic patients occurred 24 to 48 hours prior to the end of the plotted period. This represents a substantial time window during which sepsis could potentially be diagnosed and treated earlier. Given that previous studies have shown increased mortality with each hour of delay in initiation of antibiotics in patients with sepsis [3], technology involving real-time analysis of temperature patterns could potentially improve outcomes.

The sensitivity, specificity and reliability of body temperature pattern analysis, as reported in this study, are not sufficient to accurately diagnose early sepsis in a clinical setting. However, the evaluators in this study were untrained and were given no specific criteria on the basis of which to judge the temperature curves. Future prospective studies, with standardized temperature measurements and computer analysis of the temperature curves, may significantly improve both the accuracy and the reliability of this type of analysis. Patients with prolonged stays in the ICU, in whom a more extensive baseline temperature pattern would be available for computer-based analysis, would likely yield a result with much greater accuracy. Finally, more frequent measurements of core body temperature in ICU patients would also likely improve the sensitivity and specificity of this method.

## Conclusions

Abnormal body temperature pattern and greater temperature change in a 24-hour period were predictive of sepsis in afebrile ICU patients. Future prospective studies using standardized and objective temperature measurement and analysis, and incorporating other important clinical variables, should be performed to validate these results.

## Key messages

• Abnormal body temperature patterns, as identified by visual inspection, were predictive of hospital-acquired sepsis due to bacteremia or pneumonia in afebrile critically ill patients.

• Compared to nonseptic patients, septic patients experienced greater changes in body temperature in a 24-hour period during the 72 hours prior to their diagnosis of sepsis.

• Further prospective evaluation is needed to determine the role of body temperature patterns in the early diagnosis of sepsis.

## Abbreviations

APACHE: Acute Physiology and Chronic Health Evaluation; BAL: bronchoalveolar lavage; CI: confidence interval; IQR: 25% to 75% interquartile range; LOS: length of stay; MICU: medical intensive care unit; NSAID: nonsteroidal anti-inflammatory drug; OR: odds ratio; SD: standard deviation; SICU: surgical intensive care unit; SIRS: systemic inflammatory response syndrome; WBC: white blood cell.

## Competing interests

RSH reports receiving grant support from MedImmune, Bristol-Myers Squibb, Agennix and Aurigene. AMD, BMF and TCB declare that they have no competing interests.

## Authors' contributions

AMD designed the study, analyzed and interpreted the data and drafted the manuscript. BMF participated in study design and data interpretation and helped to draft the manuscript. TCB assisted with study design and manuscript preparation. RSH conceived the study, participated in study design and reviewed the manuscript. All authors read and approved the final manuscript for publication.
